# Pathways to health resilience: A cross-country analysis of health system response to re-emerging infectious diseases in Sub-Saharan Africa

**DOI:** 10.1371/journal.pone.0357814

**Published:** 2026-09-25

**Authors:** Stephen T. Odonkor

**Affiliations:** School of Public Service and Governance, Ghana Institute of Management and Public Administration, Accra, Ghana; World Health Organization, CONGO

## Abstract

Sub-Saharan Africa (SSA) faces recurrent threats from re-emerging infectious diseases, including Ebola virus disease, cholera, measles, Lassa fever, and Marburg virus disease. This study assessed the resilience of health systems in 25 SSA countries from 2000 to 2023 using a multidimensional resilience index and a first-order Markov state-transition framework. Country-year observations were classified as Low Resilience, Moderate Resilience, or High Resilience based on epidemiological performance, health financing, surveillance, response capacity, and recovery indicators. Resilience was heterogeneous and path-dependent. Low-Resilience observations had an 82.4% probability of remaining in that state in the subsequent year and only a 3.1% probability of moving directly to High Resilience. Moderate-Resilience observations had a 19.1% probability of transitioning to High Resilience and an 18.2% probability of declining to Low Resilience. High-Resilience observations had a 63.6% probability of remaining High Resilience, but a combined 36.4% probability of regression. Multinomial logistic regression showed that health expenditure per capita, domestic health-financing share, and GDP per capita were positively associated with High Resilience, whereas internal armed conflict was negatively associated with High Resilience. Southern and Eastern Africa showed stronger resilience profiles than Western and Central Africa, while Central Africa had the greatest concentration of Low-Resilience countries. These findings indicate that resilience is dynamic, unevenly distributed, and strongly shaped by sustained financing, economic capacity, and political stability. Policy priorities include strengthening domestic health financing, targeting Moderate-Resilience countries for accelerated improvement, supporting conflict-affected systems, and harmonising regional surveillance and emergency-response mechanisms.

## 1. Introduction

Infectious diseases remain a major threat to global health security, particularly in low- and middle-income settings where constrained health-system capacity and socioeconomic vulnerabilities can amplify their effects. Sub-Saharan Africa (SSA) continues to experience a high burden of endemic infectious diseases alongside recurrent epidemics of re-emerging infectious diseases (REIDs). Re-emerging infectious diseases are infections that had previously declined or been brought under relative control but have subsequently increased in incidence, severity, or geographical distribution. Important examples in SSA include measles, cholera, yellow fever, Lassa fever, Ebola virus disease, and Marburg virus disease [1–3]. The 2014–2016 Ebola epidemic in West Africa demonstrated that infectious disease outbreaks can have consequences extending beyond population health to affect national economies, social stability, and regional security [3].

The re-emergence and spread of infectious diseases in SSA are driven by interconnected environmental, demographic, socioeconomic, and health-system factors. Climate variability, environmental degradation, land-use changes, and increased human–animal interaction can alter the distribution of disease vectors and increase the risk of zoonotic transmission. At the same time, rapid population growth, unplanned urbanization, population displacement, porous national borders, political instability, and deterioration of health infrastructure can facilitate disease transmission and complicate outbreak control [4]. Weak surveillance and reporting systems, fragmented health financing, shortages of trained health personnel, and sustained underinvestment in public-health infrastructure further limit the capacity of countries to detect and contain outbreaks at an early stage [5,6]. Evidence from Nigeria, for example, has shown that weaknesses in epidemic reporting and surveillance can delay the communication of outbreak information and undermine timely public-health action [6].

These structural challenges are compounded by antimicrobial resistance, vaccine hesitancy, interruptions in routine immunization, and declining or unpredictable external health assistance. Disruptions to essential health services can reverse previous disease-control gains and create immunity gaps within populations. In several SSA countries, declining or uneven vaccination coverage has contributed to recurrent measles outbreaks, particularly in settings affected by conflict, displacement, and limited access to routine health services [7]. Cholera also remains a persistent public-health challenge in many African countries, especially where access to safe water, sanitation, hygiene services, and timely clinical care is inadequate [8]. These recurring outbreaks demonstrate that reductions in disease incidence do not necessarily indicate that the underlying health systems have developed the capacity to prevent, absorb, and recover from future health shocks.

Health-system resilience generally refers to the capacity of a health system to anticipate, prepare for, absorb, respond to, adapt to, and recover from shocks while continuing to provide essential health services [9]. Resilience therefore extends beyond the availability of hospitals, laboratories, medical supplies, and health workers. It also includes the ability to coordinate institutions, mobilize resources, maintain surveillance and service delivery, communicate effectively with communities, and learn from previous emergencies. From a dynamic resilience perspective, countries may move between different levels of resilience as their epidemiological conditions, health financing, surveillance capabilities, workforce capacity, and exposure to external shocks change over time. Resilience should therefore be understood as a changing system condition rather than a permanent national attribute.

Despite growing interest in health-system resilience, much of the existing literature has focused on individual outbreaks, specific countries, or descriptive assessments of preparedness. Relatively limited evidence exists on how resilience states evolve across SSA over extended periods, how persistent these states are, and which measurable factors are associated with transitions from lower to higher resilience. Cross-country comparisons are also frequently constrained by the absence of multidimensional measures that jointly incorporate preparedness, surveillance, outbreak outcomes, response capacity, recovery, and health-financing sustainability.

This study addresses these gaps by assessing health-system resilience to re-emerging infectious diseases across 25 SSA countries from 2000 to 2023. A multidimensional resilience index is used to classify country-year observations into Low Resilience, Moderate Resilience, and High Resilience states. A state-transition framework is then applied to estimate the probability that countries remain within or move between these resilience states over time. The analysis also examines whether measurable economic, health-financing, demographic, and conflict-related factors are associated with transitions to higher resilience states. Broader factors such as governance, institutional trust, corruption, health-insurance coverage, and community participation may also influence resilience; however, these factors are not directly measured in the present model and are therefore considered contextual issues and areas for future research rather than tested determinants.

Specifically, the study seeks to: (1) construct a multidimensional index of resilience to re-emerging infectious diseases; (2) classify the selected SSA countries according to their resilience states; (3) estimate the persistence of and transitions between Low, Moderate, and High Resilience states; (4) identify measurable factors associated with transitions to higher resilience; and (5) examine regional variations in resilience patterns. By identifying persistent vulnerabilities, transition opportunities, and potentially modifiable factors, the study aims to support more targeted and sustainable approaches to health-system strengthening and infectious disease preparedness in SSA.

## 2. Methodology

### 2.1. Study design and analytical approach

This study employed a longitudinal ecological design to examine changes in health-system resilience to re-emerging infectious diseases across 25 Sub-Saharan African countries from 2000 to 2023. The unit of analysis was the country-year. The analytical approach combined the construction of a multidimensional resilience index, classification of country-year observations into resilience states, estimation of transition probabilities between those states, and multinomial logistic regression analysis of factors associated with movement to higher resilience states.

The analysis proceeded in four stages. First, epidemiological, health-system capacity, surveillance, response, recovery, and health-financing indicators were assembled from international secondary data sources. Second, the indicators were standardised and combined using principal component analysis to produce a composite resilience index. Third, country-year observations were classified into Low Resilience, Moderate Resilience, and High Resilience states. Finally, a first-order Markov state-transition framework was used to estimate the probability of remaining in or moving between resilience states over time. Multinomial logistic regression was subsequently used to examine the measurable factors associated with transitions between states.

### 2.2. Theoretical and conceptual framework

The study was informed by health-system resilience theory, which conceptualises resilience as the capacity of a health system to anticipate, absorb, adapt to, recover from, and transform in response to shocks while continuing to provide essential health services [9–11]. Health-system resilience is therefore not limited to the availability of hospitals, laboratories, health workers, or medical supplies. It also encompasses the system’s capacity to mobilise resources, coordinate institutions, maintain surveillance and service delivery, respond to changing conditions, and learn from previous emergencies [9,10].

Consistent with systems-thinking approaches, resilience was treated as a dynamic rather than a fixed national characteristic. A country may move between different resilience states as its exposure to outbreaks, health-system sensitivity, adaptive capacity, financing arrangements, and external conditions change over time [10,11]. Previous cross-country research in the WHO African Region has similarly demonstrated that health-system resilience is multidimensional and reflects both inherent system capacity and emergency preparedness and response capabilities [5].

The conceptual framework incorporated three interrelated dimensions:

Exposure referred to the frequency and intensity of re-emerging infectious disease events experienced by a country. This dimension included the occurrence and magnitude of outbreaks of Ebola virus disease, cholera, measles, Marburg virus disease, and Lassa fever.

Sensitivity represented the extent to which a country experienced adverse consequences following an outbreak. It was reflected by indicators such as case fatality rates, outbreak duration, disease burden, and disruption of routine health services.

Adaptive capacity represented the ability of the health system to detect, respond to, contain, recover from, and learn from infectious disease outbreaks. It included surveillance capacity, laboratory availability, health-workforce capacity, emergency response systems, domestic health financing, and restoration of routine services.

**Fig 1:**
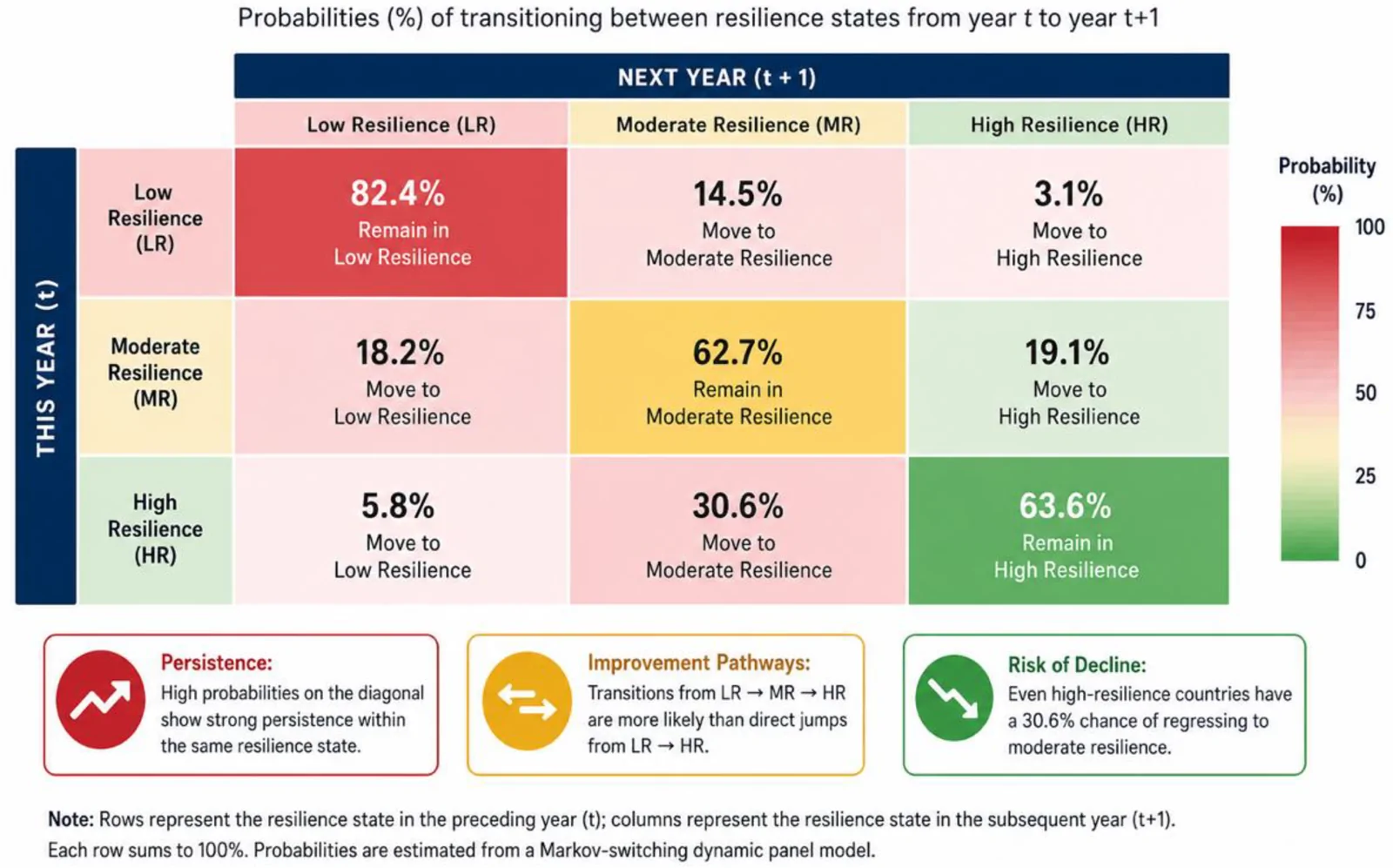
Annual resilience-state transition matrix. Rows indicate the resilience state in year t, and columns indicate the resilience state in year t + 1. Cell values are estimated annual transition probabilities, and each row sums to 100%. LR, Low Resilience; MR, Moderate Resilience; HR, High Resilience.

The framework assumed that countries facing high exposure and sensitivity but possessing limited adaptive capacity would be more likely to remain in a Low Resilience state. Conversely, countries with stronger surveillance, response, financing, and recovery capacity would have a greater probability of occupying or transitioning towards a High Resilience state. Fig 1 presents the conceptual relationships among exposure, sensitivity, adaptive capacity, resilience classification, and state transitions.

### 2.3. Study setting, country selection, and period of analysis

The study covered 25 countries in Sub-Saharan Africa over the period 2000–2023. Countries were included based on the availability of sufficiently complete and comparable data for the variables required to construct the resilience index and estimate the transition models.

The selection was intended to capture variation in epidemiological burden, health-system capacity, economic development, health financing, and exposure to external shocks. The sample therefore included relatively stronger-performing health systems as well as conflict-affected and institutionally fragile settings.

Although the study period extended from 2000 to 2023, not all data sources provided annual observations throughout this entire period. For example, Joint External Evaluation assessments were introduced after the beginning of the study period and were not available annually for every country. Such measures were therefore used only for the years in which comparable observations were available or were complemented with annual indicators from the International Health Regulations monitoring framework. The procedures used to manage missing observations, non-annual indicators, and differences in data coverage are described in Section 2.5.

### 2.4. Data sources

Data were compiled from internationally recognised databases and institutional reports. The use of multiple sources enabled the study to incorporate epidemiological outcomes, health-system capacity, financing, surveillance, and external shocks. Table 1 summarises the principal data domains and sources.

**Table 1:** Data sources and indicators used in the analysis.

Data domain	Primary source	Selected indicators
Health-system capacity	WHO Global Health Observatory	Health-worker density, hospital-bed density, immunisation coverage and selected service-capacity indicators
Disease incidence and mortality	Institute for Health Metrics and Evaluation Global Burden of Disease database; WHO and Africa CDC outbreak reports	Incidence, mortality and case fatality associated with Ebola, cholera, measles, Marburg virus disease and Lassa fever
Health financing	World Bank World Development Indicators; WHO Global Health Expenditure Database	Current health expenditure per capita, domestic government health expenditure, external health expenditure and health expenditure as a proportion of GDP
Surveillance and preparedness	WHO International Health Regulations monitoring framework; Joint External Evaluation reports	Surveillance capacity, laboratory systems, emergency preparedness and response capacity
Socioeconomic characteristics	World Bank World Development Indicators	GDP per capita, urban population and population density
Conflict and external shocks	Uppsala Conflict Data Program or ACLED; EM-DAT	Internal armed conflict, disasters, displacement and climate-related emergencies

Where the same indicator was available from more than one source, preference was given to the source with the widest temporal and geographical coverage and the most consistent definition across countries.

### 2.5. Data preparation and management of missing observations

Data from the different sources were merged using country and year identifiers. Country names and codes were harmonised before merging. Variables expressed in monetary terms were converted to constant prices or purchasing-power-adjusted values where appropriate to improve comparability over time.

The percentage of missing observations was assessed for each variable and country. Variables with extensive missingness or major inconsistencies in definition were excluded from the composite index. Country-year observations lacking the minimum data required to calculate the index were treated as missing and excluded from the relevant analysis.

### 2.6. Selection of resilience indicators

Indicators were selected on the basis of health-system resilience theory, empirical studies of health-system resilience in Africa, relevance to infectious disease preparedness and response, availability across countries, and comparability over time [5,9–11]. The resilience index developed in this study was not adopted unchanged from a previously published index. Instead, it was constructed for the present analysis using established principles for developing composite indicators and previous health-system resilience frameworks [5,10,12].

The indicators were organised into four operational domains.

#### 2.6.1 Preparedness and Surveillance.

This domain reflected the capacity to identify and verify outbreaks promptly. Candidate indicators included:

- time from outbreak onset to detection;
- surveillance-system capacity;
- laboratory capacity or laboratory density;
- immunisation coverage;
- surveillance or preparedness expenditure, where consistently available; and
- relevant International Health Regulations capacity scores.

#### 2.6.2 Response Effectiveness.

This domain assessed the ability to reduce illness, mortality, and transmission after an outbreak had been detected. Candidate indicators included:

- outbreak-specific case fatality rate;
- health-workforce density or surge capacity;
- emergency response capacity;
- community-based surveillance or risk-communication capacity; and
- time from detection to initiation of response measures.

#### 2.6.3 Recovery and service continuity.

This domain represented the ability to restore affected services and maintain essential health functions. Candidate indicators included:

- duration from outbreak declaration to closure;
- restoration of routine immunisation and essential services;
- post-outbreak investment in health infrastructure; and
- continuity of essential service-delivery indicators.

#### 2.6.4 Health-financing sustainability.

This domain captured the availability, domestic ownership, and stability of resources required to sustain preparedness and response. Candidate indicators included:

- current health expenditure per capita;
- domestic government share of health expenditure;
- external health-financing share;
- health expenditure as a proportion of GDP; and
- variation in annual government health expenditure.

### 2.7. Construction of the composite resilience index

All retained indicators were coded so that higher values represented stronger resilience. Adverse indicators, including case fatality, detection delay, outbreak duration, and external funding dependence where applicable, were reverse-coded. Continuous indicators were standardised using z-scores to place variables measured on different scales on a comparable metric. Principal component analysis was then applied to the standardised indicators. Component retention was guided by eigenvalues, the scree plot, conceptual coherence, and the proportion of variance explained. Indicator weights were derived from the retained component loadings, and the weighted scores were rescaled to range from 0 to 1, with higher scores indicating greater health-system resilience.

### 2.8. Classification of resilience states

Country-year resilience scores were classified using study-defined thresholds: Low Resilience for scores below 0.25, Moderate Resilience for scores from 0.25 to below 0.60, and High Resilience for scores of 0.60 or higher. These thresholds were selected to distinguish persistent vulnerability, intermediate system capacity, and comparatively strong resilience. They should not be described as quartiles. Sensitivity analyses evaluated whether the findings were robust to alternative classification rules, including tertile-based and equal-width thresholds.

### 2.9. First-order Markov state-transition analysis

Let S_it denote the observed resilience state of country i in year t, where S_it ∈ {LR, MR, HR}. A first-order Markov state-transition model was used to estimate P(S_it = j | S_i,t − 1 = s), the probability that a country-year observation moved from state s in the preceding year to state j in the subsequent year. The transition matrix was estimated from observed annual state sequences, and each row was constrained to sum to 1. This approach describes state persistence and movement; it is not a Markov-switching latent-regime model.

### 2.10. Multinomial logistic regression

Multinomial logistic regression was used to examine country-level factors associated with resilience classification, with Low Resilience specified as the reference outcome. Explanatory variables included health expenditure per capita, domestic health-financing share, GDP per capita, urbanisation or urban population density, and internal armed conflict. Results were reported as adjusted odds ratios with 95% confidence intervals and p-values. Standard errors were clustered at the country level to account for repeated observations within countries.

### 2.11. Model diagnostics and robustness analyses

Multicollinearity was assessed using correlation coefficients and variance inflation factors, with values below 5 interpreted as indicating no serious multicollinearity. Model fit was evaluated using likelihood-ratio tests, the Akaike information criterion, the Bayesian information criterion, and comparison of predicted and observed resilience classifications. Robustness analyses included alternative resilience thresholds, exclusion of the COVID-19 years, leave-one-country-out estimation, complete-case analysis, alternative specifications of financing and urbanisation, models excluding conflict-affected countries, and alternative lag structures. Only analyses actually estimated should be reported in the final supplementary results.

### 2.12. Ethical considerations and data availability

The study used publicly available, aggregate secondary data and did not involve human participants or individually identifiable information. Formal informed consent was therefore not applicable. Data were managed in accordance with open-science and research-integrity principles. The harmonised analytical dataset and code should be made available in a public repository or from the corresponding author, subject to the reuse conditions of the original data providers.

## 3. Results

### 3.1. Country coverage and distribution of resilience states

The analysis included 25 Sub-Saharan African countries observed over the study period. Based on the composite Health-System Resilience Index, 8 countries were classified as Low Resilience, 12 as Moderate Resilience, and 5 as High Resilience. As shown in Table 2, Moderate Resilience was the most common classification, accounting for 48.0% of the countries. Low-Resilience countries represented 32.0% of the sample, while High-Resilience countries accounted for 20.0%.

**Table 2:** Distribution of countries by resilience state.

Resilience state	Number of countries	Percentage
Low Resilience	8	32.0
Moderate Resilience	12	48.0
High Resilience	5	20.0
**Total**	**25**	**100.0**

The countries classified as High Resilience were Rwanda, Botswana, Ghana, Namibia, and Mauritius. Countries explicitly identified within the Moderate-Resilience group included Nigeria, Kenya, Senegal, and Tanzania. South Sudan, the Central African Republic, Somalia, Guinea-Bissau, and the Democratic Republic of Congo were among the countries classified as Low Resilience.

### 3.2. Regional variation in health-system resilience

Marked differences were observed across the four Sub-Saharan African subregions. As presented in Table 3, Southern Africa recorded the highest proportion of High-Resilience countries, at 40%, followed by Eastern Africa at 36%. Western Africa had 20% of its included countries classified as High Resilience, while no country from Central Africa was classified in the High-Resilience category.

**Table 3:** Regional distribution of resilience classifications.

Subregion	High Resilience (%)	Moderate Resilience (%)	Low Resilience (%)
Eastern Africa	36	44	20
Southern Africa	40	40	20
Western Africa	20	50	30
Central Africa	0	38	62

Central Africa exhibited the greatest concentration of Low-Resilience countries, with 62% classified as Low Resilience. The corresponding proportions were 30% in Western Africa and 20% each in Eastern and Southern Africa (Table 3).

Overall, the regional distribution shown in Table 3 indicates substantial heterogeneity in health-system resilience. Southern and Eastern Africa contained a greater proportion of countries with comparatively stronger resilience, whereas Central Africa was characterised by a predominance of Low-Resilience states.

### 3.3. Resurgence patterns of selected infectious diseases

The descriptive outbreak analysis showed considerable variation in disease severity and detection delays across the selected re-emerging infectious diseases. As shown in Table 4, Marburg virus disease had the highest reported average case fatality rate, at 82.0%, followed by Ebola virus disease at 39.0% and Lassa fever at 18.0%. Cholera and measles had lower reported average case fatality rates of 2.9% and 1.3%, respectively.

**Table 4:** Selected disease-resurgence indicators.

Disease	Selected countries	Period represented	Average case fatality rate (%)	Average detection delay
Ebola virus disease	Democratic Republic of Congo, Guinea and Sierra Leone	2014–2016 and 2018–2022	39.0	21 days
Cholera	Malawi, Mozambique and Nigeria	2010–2023	2.9	28 days
Measles	Ethiopia, Nigeria and Democratic Republic of Congo	2018–2021	1.3	41 days
Marburg virus disease	Equatorial Guinea and Tanzania	2023	82.0	17 days
Lassa fever	Nigeria and Liberia	2017–2023	18.0	33 days

The detection-delay results in Table 4 showed that measles had the longest average detection delay, at 41 days, followed by Lassa fever at 33 days and cholera at 28 days. Ebola virus disease had an average detection delay of 21 days, while Marburg virus disease had the shortest reported detection delay, at 17 days.

The findings in Table 4 indicate that disease severity and detection performance did not follow the same pattern. Marburg virus disease had the highest case fatality rate but the shortest reported detection delay, whereas measles had the lowest case fatality rate but the longest detection delay.

### 3.4. Persistence and transition between resilience states

The estimated annual transition matrix showed that countries were generally more likely to remain in their existing resilience state than to move to another state. The complete transition probabilities are presented in Table 5.

**Table 5:** Annual resilience-state transition matrix.

Previous resilience state	Low Resilience	Moderate Resilience	High Resilience
Low Resilience	82.4%	14.5%	3.1%
Moderate Resilience	18.2%	62.7%	19.1%
High Resilience	5.8%	30.6%	63.6%

A country classified as Low Resilience had an 82.4% probability of remaining in that state in the following year. Its probability of moving to Moderate Resilience was 14.5%, while the probability of moving directly to High Resilience was only 3.1% (Table 5).

Countries in the Moderate-Resilience state had a 62.7% probability of remaining Moderate. The probability of deterioration from Moderate to Low Resilience was 18.2%, while the probability of improvement from Moderate to High Resilience was 19.1% (Table 5).

Countries classified as High Resilience had a 63.6% probability of remaining in the High-Resilience state. However, they had a 30.6% probability of declining to Moderate Resilience and a 5.8% probability of falling to Low Resilience (**Table 5**).

The transition probabilities in Table 5 demonstrate that Low Resilience was the most persistent state. Movement directly from Low to High Resilience was uncommon, suggesting that improvements in national resilience were more likely to occur gradually through the Moderate-Resilience state.

The transition matrix also indicates that High Resilience was not permanent. Although High-Resilience countries had a 63.6% probability of remaining in that state, nearly one-third had a probability of declining to Moderate Resilience in the subsequent year (Table 5).

It is important to note that the probability of transition from Moderate to High Resilience was 19.1%. The value of 30.6% shown in Table 5 represents transition from High to Moderate Resilience and should not be interpreted as improvement from Moderate to High Resilience.

### 3.5. Resilience-state transition probabilities

Fig 1 presents the estimated probabilities of countries remaining in or transitioning between Low Resilience, Moderate Resilience, and High Resilience states. The transition matrix indicates substantial persistence within each resilience category, particularly among countries classified as Low Resilience.

Countries in the Low-Resilience state had an 82.4% probability of remaining in that state during the subsequent period. The probability of moving from Low to Moderate Resilience was 14.5%, while direct movement from Low to High Resilience was uncommon, at 3.1% (Fig 1). These findings indicate that countries with weak resilience were more likely to remain in a condition of persistent vulnerability than to experience rapid improvement.

Countries classified as Moderate Resilience had a 62.7% probability of remaining in that state. Their probability of moving to High Resilience was 19.1%, while the probability of declining to Low Resilience was 18.2% (Fig 1). This suggests that Moderate-Resilience countries had nearly equal probabilities of improvement and deterioration.

High-Resilience countries had a 63.6% probability of remaining in the High-Resilience state. However, the probability of declining from High to Moderate Resilience was 30.6%, while the probability of falling directly to Low Resilience was 5.8% (Fig 1). Thus, although High Resilience was relatively persistent, it was not irreversible.

Overall, the transition patterns demonstrate that health-system resilience was path-dependent but dynamic. Movement directly from Low to High Resilience was rare, suggesting that resilience improvement was more likely to occur gradually through the Moderate-Resilience state. The observed probability of decline from High to Moderate Resilience further indicates that resilience gains require continued maintenance and investment.

### 3.6. Factors associated with high resilience

Multinomial logistic regression was used to identify country-level factors associated with High Resilience relative to Low Resilience. The complete regression estimates are presented in Table 6.

**Table 6:** Multinomial logistic regression estimates for High versus Low Resilience.

Predictor	Coefficient	Standard error	Odds ratio	95% confidence interval	p-value
Health expenditure per capita	0.014	0.004	1.014	1.006–1.022	<0.001
Domestic health-financing share	0.009	0.003	1.009	1.003–1.015	0.003
Internal armed conflict	−0.221	0.052	0.802	0.724–0.888	<0.001
Urbanisation/urban density	0.005	0.003	1.005	0.999–1.011	0.096
GDP per capita	0.017	0.005	1.017	1.007–1.027	0.001

Health expenditure per capita, domestic health financing, and GDP per capita were positively associated with High Resilience. Internal armed conflict was negatively associated with High Resilience. Urbanisation or urban density showed a positive but statistically non-significant association (Table 6).

#### 3.6.1. Health expenditure per capita.

As shown in Table 6, health expenditure per capita was positively associated with High Resilience. Each one-unit increase in health expenditure per capita was associated with a 1.4% increase in the odds of being in the High- rather than Low-Resilience state, holding the other variables constant:


$$\begin{array}{c} \left[\frac{\left(1.014-1\right)}{\text{times\textasciitilde{}}100}=1.4\%\right] \end{array}$$

The association was statistically significant, with an odds ratio of 1.014, a 95% confidence interval of 1.006–1.022, and p < 0.001 (Table 6).

The substantive interpretation of this result depends on the unit in which health expenditure was entered into the regression model.

#### 3.6.2. Domestic health financing.

The domestic share of health financing was also positively associated with High Resilience. As reported in **Table 6**, a one-unit increase in domestic financing was associated with approximately 0.9% higher odds of High rather than Low Resilience:


$$\begin{array}{c} \left[\frac{\left(1.009-1\right)}{\text{times\textasciitilde{}}100}=0.9\%\right] \end{array}$$

The association was statistically significant, with an odds ratio of 1.009, a 95% confidence interval of 1.003–1.015, and p = 0.003 (Table 6).

Where the variable was measured in percentage points, the estimate indicates that each one-percentage-point increase in the domestic share of health financing was associated with a modest increase in the odds of High Resilience.

#### 3.6.3. Internal armed conflict.

Internal armed conflict was negatively associated with High Resilience. Countries experiencing internal armed conflict had approximately 19.8% lower odds of being classified as High rather than Low Resilience:


$$\begin{array}{c} \left[\frac{1-0.802)}{\text{times\textasciitilde{}}100}=19.8\%\right] \end{array}$$

As shown in Table 6, the association was statistically significant, with an odds ratio of 0.802, a 95% confidence interval of 0.724–0.888, and p < 0.001.

#### 3.6.4. Urbanisation or Urban Density.

Urbanisation or urban density had a positive but statistically non-significant association with High Resilience. The estimated odds ratio was 1.005, with a 95% confidence interval of 0.999–1.011 and p = 0.096 (Table 6).

Because the confidence interval included 1 and the p-value exceeded 0.05, this variable was not considered a statistically significant independent predictor of High Resilience.

#### 3.6.5. GDP per capita.

GDP per capita was positively associated with High Resilience. As reported in Table 6, the odds ratio was 1.017, with a 95% confidence interval of 1.007–1.027 and p = 0.001.

This corresponds to an estimated 1.7% increase in the odds of High rather than Low Resilience for each one-unit increase in GDP per capita:


$$\begin{array}{c} \left[\frac{\left(1.017-1\right)}{\text{times\textasciitilde{}}100}=1.7\%\right] \end{array}$$

The practical magnitude of this association depends on the unit and transformation used for GDP per capita.

### 3.7. Relative importance of the predictors

Among the variables presented in Table 6, internal armed conflict demonstrated the largest adverse association with High Resilience. Health expenditure per capita, domestic health financing, and GDP per capita showed positive associations, although their odds ratios were numerically close to 1 because they were estimated per one-unit increase.

The odds ratios in Table 6 should therefore not be compared solely according to their distance from 1 without considering the measurement scale of each predictor. Reporting the effects for more meaningful increments would improve interpretation.

### 3.8. Summary of principal findings

The principal findings are summarised across Tables 2–6.

First, almost half of the countries were classified as Moderate Resilience, while only one-fifth achieved High Resilience (Table 2).

Second, resilience varied substantially by subregion. Southern and Eastern Africa contained the highest proportions of High-Resilience countries, whereas Central Africa had the greatest concentration of Low-Resilience states (Table 3).

Third, the selected infectious diseases differed substantially in case fatality rates and detection delays (Table 4).

Fourth, resilience states were persistent over time. Low-Resilience countries had an 82.4% probability of remaining Low Resilience, while movement directly from Low to High Resilience was rare, at 3.1%. Moderate-Resilience countries had a 19.1% probability of moving to High Resilience (Table 5).

Finally, higher health expenditure, stronger domestic financing, and higher GDP per capita were associated with increased odds of High Resilience. Internal armed conflict was associated with reduced odds of High Resilience, while urbanisation or urban density was not statistically significant at the conventional 5% level (Table 6).

## 4. Discussion

### 4.1. Distribution of health-system resilience across Sub-Saharan Africa

This study provides one of the few longitudinal, cross-country assessments of health-system resilience against re-emerging infectious diseases in Sub-Saharan Africa (SSA). The findings indicate substantial heterogeneity in resilience across the region, with nearly half of the countries classified as having Moderate Resilience, one-third remaining in the Low-Resilience category, and only one-fifth demonstrating High Resilience. These results suggest that while many African health systems have strengthened their preparedness and response capacities over the past two decades, considerable disparities remain in their ability to absorb, adapt to, and recover from recurrent public health emergencies. The observed distribution is consistent with recent regional assessments by the WHO Regional Office for Africa, which demonstrated wide variation in resilience capacities among Member States, particularly in governance, resource mobilization, service diversity, and adaptive capacity.13 The findings also support the growing consensus that resilience should be understood as a dynamic systems property rather than a static measure of health-system performance [5,13].

The predominance of Moderate-Resilience countries likely reflects the considerable investments made in disease surveillance, laboratory networks, emergency preparedness, and Integrated Disease Surveillance and Response (IDSR) following major outbreaks such as Ebola virus disease, COVID-19, cholera, yellow fever, and mpox. Since the 2014–2016 West African Ebola epidemic, many SSA countries have strengthened national public health institutes, emergency operations centres, laboratory diagnostic capacity, and workforce training through support from the World Health Organization (WHO), Africa Centres for Disease Control and Prevention (Africa CDC), and other development partners.15–17 These reforms have improved countries’ ability to detect and respond to outbreaks while maintaining essential health services during public health emergencies. Nevertheless, the persistence of Moderate rather than High Resilience indicates that these improvements have not yet translated into uniformly robust and sustainable health systems across the region. This observation is consistent with WHO regional reports highlighting that many countries continue to experience deficiencies in financing, governance, human resources for health, and supply-chain management despite improvements in surveillance and emergency preparedness [14–16].

The finding that only a small proportion of countries achieved High Resilience is unsurprising given the complex and multidimensional nature of health-system resilience. High-performing countries in this study—including Rwanda, Botswana, Ghana, Namibia, and Mauritius—have consistently demonstrated stronger investments in primary health care, universal health coverage (UHC), health information systems, and domestic health financing than many of their regional counterparts. Previous studies have shown that resilient health systems are characterised not only by the availability of health resources but also by effective governance, institutional learning, flexible service delivery, and the capacity to reorganise during crises while maintaining essential health services.13,18,19 These characteristics align closely with the conceptual framework underpinning the present study and help explain why relatively few countries attained the highest resilience category despite improvements in outbreak preparedness across the continent [5,9,10].

Conversely, countries classified as Low Resilience were predominantly those experiencing prolonged conflict, political instability, chronic underinvestment in health systems, or recurrent humanitarian emergencies. Fragile health systems often face simultaneous shortages of skilled health workers, inadequate laboratory capacity, weak surveillance systems, interrupted medicine supply chains, and limited domestic fiscal space, all of which reduce their ability to respond effectively to infectious disease outbreaks. Similar patterns have been documented in studies examining health-system performance in fragile and conflict-affected settings, where repeated shocks overwhelm already constrained health systems and undermine long-term recovery.18–21 The concentration of Low-Resilience countries among fragile states therefore reinforces the close interrelationship between governance, political stability, economic development, and health security. Rather than representing isolated weaknesses within the health sector, low resilience appears to reflect broader systemic vulnerabilities affecting national institutions and public administration [9,10,17].

Compared with previous resilience assessments conducted in the WHO African Region, the present study extends the existing evidence by incorporating longitudinal transition analysis rather than relying solely on cross-sectional resilience scores.13 While earlier studies have primarily focused on measuring resilience capacities at a single point in time, the present analysis demonstrates that resilience should also be viewed as a temporal process characterised by gradual improvement, persistence, or decline. This dynamic perspective provides a more comprehensive understanding of how health systems evolve following repeated infectious disease threats and offers additional insights for policymakers seeking to strengthen preparedness over the long term. The findings therefore contribute to the emerging literature advocating for resilience measurement frameworks that integrate preparedness, response, recovery, and adaptive learning within a single analytical approach [5,13].

### 4.2. Regional differences in health-system resilience

Marked regional differences in health-system resilience were observed across Sub-Saharan Africa, with Southern Africa recording the highest proportion of High-Resilience countries, whereas Central Africa had the greatest concentration of Low-Resilience countries. These findings are consistent with recent assessments by the WHO Regional Office for Africa, which demonstrate substantial heterogeneity in health-system resilience across Member States, driven by differences in governance, health financing, service delivery, emergency preparedness, and institutional capacity.17,18 The WHO African Region’s Framework for Sustaining Resilient Health Systems further emphasizes that countries across the region face differing capacities to prevent, detect, and respond to health emergencies because of long-standing disparities in health-system investment, workforce availability, infrastructure, and governance [16,17].

The relatively stronger resilience observed in Southern Africa may reflect decades of sustained investment in health systems through HIV/AIDS, tuberculosis, and malaria control programmes, which have strengthened laboratory networks, surveillance systems, pharmaceutical supply chains, health information systems, and workforce development. Countries such as Botswana and Namibia have consistently expanded domestic financing for health, improved primary healthcare delivery, and strengthened public health institutions, enabling them to respond more effectively to successive infectious disease threats. Similar observations have been reported in comparative analyses of health-system resilience across the WHO African Region, where countries with stronger institutional capacity and diversified health services demonstrated greater adaptive and transformative capacities during public health emergencies [5,14].

In contrast, the predominance of Low-Resilience countries within Central Africa is likely attributable to the cumulative effects of prolonged armed conflict, political instability, weak governance, population displacement, and chronic underinvestment in public health infrastructure. Fragile and conflict-affected settings often experience repeated disruptions to health service delivery, destruction of health facilities, shortages of skilled personnel, interrupted medicine supply chains, and weakened disease surveillance systems. A recent systematic scoping review of health-system resilience in fragile and conflict-affected settings found that these interconnected challenges substantially undermine countries’ abilities to prepare for, absorb, and recover from public health shocks.19 These findings align closely with the present study, where conflict emerged as one of the strongest determinants of reduced resilience [17].

The regional variation observed in this study also highlights the importance of broader socioeconomic and governance contexts beyond health-sector investments alone. Countries with stronger public institutions, better fiscal capacity, and more stable political environments are generally better positioned to sustain essential health services during crises while simultaneously responding to emerging outbreaks. Conversely, countries experiencing recurrent humanitarian crises frequently allocate scarce public resources toward emergency response rather than long-term health-system strengthening, thereby perpetuating structural vulnerabilities. This interpretation is consistent with resilience frameworks that conceptualize health systems as complex adaptive systems whose performance depends on interactions among governance, financing, leadership, community engagement, and multisectoral coordination rather than individual health-sector inputs alone [5,10,16].

Interestingly, the present findings differ slightly from several earlier cross-sectional assessments that ranked countries primarily according to preparedness capacities measured through Joint External Evaluations (JEEs) or International Health Regulations (IHR) indicators. While preparedness assessments provide valuable information regarding technical capacities, they often capture only a snapshot of system readiness and may not fully reflect how health systems perform during repeated or prolonged shocks. By incorporating longitudinal transition analysis alongside multidimensional resilience indicators, the present study demonstrates that resilience encompasses not only preparedness but also the ability to maintain essential services, adapt to evolving threats, recover after crises, and continuously strengthen institutional capacity over time. This broader conceptualisation is increasingly advocated by WHO and contemporary resilience literature, which recognises resilience as a dynamic process rather than a fixed outcome [5,16].

These findings have important policy implications for regional health security. First, strengthening resilience should extend beyond emergency preparedness to include sustained investments in primary healthcare, domestic health financing, workforce retention, governance reforms, and resilient supply-chain systems. Second, countries with lower resilience would benefit from greater regional collaboration through mechanisms coordinated by the Africa Centres for Disease Control and Prevention (Africa CDC), including cross-border surveillance, laboratory networking, workforce development, and coordinated emergency response. Finally, implementation of the revised WHO African Region Framework for Sustaining Resilient Health Systems (2023–2030) provides an important opportunity for Member States to integrate universal health coverage, health security, and emergency preparedness within a single policy agenda rather than treating them as separate priorities.18 Such integrated approaches are likely to reduce regional disparities and strengthen collective preparedness for future epidemics and other public health emergencies [16,24,25].

### 4.3. Persistence and transition of health-system resilience states

A notable contribution of this study is the examination of resilience as a dynamic process through the application of a first-order Markov state-transition model. Unlike conventional cross-sectional resilience assessments that provide a snapshot of health-system performance, the transition analysis revealed how countries moved between resilience states over time. The results demonstrated substantial persistence across all resilience categories, with countries classified as Low Resilience exhibiting an 82.4% probability of remaining in that state during the subsequent period. Similarly, Moderate- and High-Resilience countries had probabilities of 62.7% and 63.6%, respectively, of remaining within their existing resilience categories (Table 5; Fig 1). These findings suggest that health-system resilience is characterized by considerable structural inertia, whereby both strengths and weaknesses tend to persist over time rather than change rapidly [9–11].

The high persistence observed among Low-Resilience countries indicates that resilience deficits are deeply embedded within broader institutional, financial, and governance structures. Countries with weak health systems frequently experience chronic shortages of skilled health workers, inadequate infrastructure, fragmented surveillance systems, limited domestic financing, and poor governance, which collectively constrain their ability to improve preparedness and response capacities. Similar observations have been reported by Kruk et al., who argued that resilience should be viewed as the capacity of health systems to absorb shocks while maintaining core functions through adaptation and transformation rather than merely recovering after crises [20]. Likewise, Blanchet et al. emphasized that resilience emerges from interactions among governance, learning, flexibility, and institutional capacity, making rapid improvements unlikely where these foundational components remain weak [21]. These conceptual frameworks provide a plausible explanation for the strong persistence of Low Resilience observed in the present study [9,10].

The rarity of direct transitions from Low to High Resilience (3.1%) further reinforces the notion that resilience development is generally incremental rather than transformational. Most improvements occurred through progression from Low to Moderate Resilience before reaching higher levels of system performance. This finding aligns with the adaptive systems perspective, which proposes that resilient health systems evolve through continuous learning, institutional reform, and cumulative investments rather than isolated interventions or emergency responses [22]. Evidence from countries recovering from the Ebola virus disease epidemic similarly demonstrated that sustained improvements in surveillance, laboratory capacity, workforce development, and emergency preparedness required many years of coordinated investment before measurable gains in resilience became apparent [23]. Consequently, expectations of rapid transformation following individual donor-funded programmes or emergency preparedness initiatives may be unrealistic unless accompanied by broader reforms in governance, financing, and service delivery [11,18].

Although High-Resilience countries demonstrated a relatively high probability (63.6%) of maintaining their resilience status, the observed 30.6% probability of transition from High to Moderate Resilience indicates that resilience is not a permanent characteristic of health systems. Instead, resilience appears to be dynamic and reversible, reflecting changing political, economic, and epidemiological conditions. Similar conclusions have been reached by recent studies examining health-system responses to the COVID-19 pandemic, which showed that even countries with historically strong health systems experienced substantial disruptions when confronted with prolonged public health emergencies, workforce shortages, supply-chain interruptions, and competing fiscal priorities [24,25]. These observations challenge the misconception that resilience represents an end state; rather, resilient systems require continuous investment, institutional learning, and adaptive governance to sustain performance during successive crises [18–20].

The persistence of resilience states observed in this study also has important implications for implementation of the International Health Regulations (2005) and the WHO Health Emergency Preparedness, Response and Resilience (HEPR) Framework. Both frameworks emphasize that preparedness should not be considered a one-time achievement but rather a continuous process of strengthening core capacities, surveillance systems, multisectoral coordination, and emergency response mechanisms. Countries remaining in the Low-Resilience category are therefore unlikely to achieve sustainable improvements through short-term emergency funding alone. Instead, long-term investments in primary health care, health workforce development, laboratory systems, domestic resource mobilization, and governance reforms are required to facilitate gradual transitions toward higher resilience states [21,22].

An important implication of the transition analysis is that resilience trajectories differ considerably across countries despite being located within the same geographic region. This finding suggests that national policy choices, institutional leadership, and domestic investment may influence resilience progression beyond structural socioeconomic constraints alone. Several countries with similar levels of economic development demonstrated markedly different resilience trajectories, implying that governance effectiveness, accountability, and strategic health-system planning may partially mediate the relationship between national wealth and resilience outcomes. Similar conclusions have been reported in comparative analyses of health-system resilience across low- and middle-income countries, where governance quality and institutional learning frequently explained variations in resilience beyond differences in health expenditure alone [5,23].

Overall, the transition analysis extends existing resilience literature by demonstrating that resilience should be conceptualized as a dynamic developmental pathway rather than a static classification. The application of longitudinal state-transition modelling provides policymakers with valuable insights into the likelihood of sustained improvement or deterioration and enables identification of countries requiring intensified long-term support. These findings reinforce growing international calls for resilience monitoring systems that track changes over time rather than relying exclusively on periodic preparedness assessments. Such approaches are consistent with recommendations from WHO and Africa CDC advocating continuous monitoring of resilience as part of broader health security and universal health coverage strategies [16,22,24].

### 4.4 Determinants of health-system resilience

The multinomial logistic regression analysis identified health expenditure per capita, domestic health financing, and gross domestic product (GDP) per capita as significant positive predictors of health-system resilience, whereas internal armed conflict was associated with substantially lower odds of achieving High Resilience. These findings reinforce the growing body of evidence demonstrating that resilient health systems are underpinned by sustained investment, strong governance, and stable socioeconomic environments rather than emergency preparedness alone [5,9,11].

Health expenditure per capita emerged as one of the strongest predictors of High Resilience. Countries allocating greater financial resources to health were significantly more likely to maintain resilient health systems capable of responding effectively to re-emerging infectious diseases. This finding is consistent with previous cross-country analyses showing that adequate health financing improves service availability, laboratory capacity, surveillance systems, emergency preparedness, and workforce retention, all of which enhance the absorptive and adaptive capacities of health systems during crises. During the COVID-19 pandemic, countries with stronger health financing mechanisms were generally better able to sustain essential health services while simultaneously responding to unprecedented public health demands [24,25]. Similarly, WHO has consistently emphasized that sustainable investment in health systems is fundamental to achieving both Universal Health Coverage (UHC) and global health security because underfunded systems frequently struggle to maintain routine services during emergencies [18–20,22].

An equally important finding was the significant contribution of domestic health financing to resilience. Countries that financed a larger proportion of their health expenditure through domestic resources demonstrated significantly greater odds of belonging to the High-Resilience category. This observation supports previous research indicating that excessive dependence on external donor funding may undermine long-term resilience because donor priorities often fluctuate according to changing global health agendas and emergency response needs. Domestic financing enables governments to institutionalize preparedness activities, maintain surveillance systems between outbreaks, strengthen emergency stockpiles, and retain skilled health workers irrespective of donor funding cycles. The Africa Leadership Meeting Declaration on Increasing Domestic Health Financing similarly advocates increased national investment as a prerequisite for sustainable health-system strengthening across Africa. The present findings therefore reinforce ongoing continental efforts to reduce dependence on external assistance while improving national ownership of health security programmes [24,25].

GDP per capita was also positively associated with High Resilience, suggesting that broader macroeconomic development contributes to stronger health-system performance. Wealthier countries generally possess larger fiscal space to invest in infrastructure, human resources for health, research, digital health technologies, and emergency preparedness. Similar associations between national income and health-system performance have been reported in global comparative analyses examining pandemic preparedness and health security capacities.35 However, the relationship observed in the present study should not be interpreted as implying that economic growth alone guarantees resilience. Several countries with comparable levels of economic development demonstrated markedly different resilience classifications, indicating that governance quality, institutional effectiveness, accountability, and efficient resource allocation remain critical determinants of health-system performance [13,20,21]. This finding is consistent with resilience frameworks that emphasize governance as the mechanism through which financial resources are translated into effective service delivery and emergency response capacity [5,10,11].

Internal armed conflict emerged as the strongest negative predictor of resilience. Countries experiencing conflict were significantly less likely to achieve High Resilience, highlighting the profound effects of insecurity on health-system functioning. Conflict disrupts health infrastructure, destroys health facilities, displaces populations and healthcare workers, interrupts medicine supply chains, weakens surveillance systems, and diverts government expenditure away from health towards security operations [19]These disruptions reduce the capacity of health systems to detect, respond to, and recover from infectious disease outbreaks while simultaneously increasing population vulnerability through displacement, food insecurity, and reduced access to essential health services. Similar findings have been reported in studies conducted across fragile and conflict-affected settings, where prolonged insecurity consistently undermined outbreak preparedness and delayed recovery following public health emergencies [19]. The persistence of Low-Resilience countries within conflict-affected regions observed in the present study therefore reflects broader structural challenges extending beyond the health sector itself [17].

Interestingly, urban population density was not independently associated with resilience after adjustment for health financing, economic development, and conflict. Although densely populated urban areas may facilitate rapid disease transmission, they also frequently possess better healthcare infrastructure, specialist services, laboratory capacity, and emergency response mechanisms than rural areas. Consequently, the effects of urbanization on resilience may operate through improvements in health-system capacity rather than population density alone. Similar conclusions have been reported by studies examining COVID-19 responses across low- and middle-income countries, where governance quality, surveillance capacity, and health-system organization explained substantially more variation in pandemic outcomes than urbanization itself [23,24]. The absence of an independent association in the present analysis therefore suggests that urbanization should be interpreted as a contextual factor whose influence depends largely on accompanying investments in health-system capacity [18,19].

Collectively, these findings have important implications for policy implementation under the WHO Health Emergency Preparedness, Response and Resilience (HEPR) Framework and the International Health Regulations (2005). Both frameworks emphasize that resilient health systems require sustained financing, multisectoral governance, robust surveillance, resilient service delivery, and effective emergency preparedness rather than isolated investments in outbreak response [26,27]. The present findings support these recommendations by demonstrating that resilience is strongly associated with long-term structural investments rather than temporary emergency interventions. Consequently, governments should prioritize domestic resource mobilization, strengthen public financial management, protect health budgets during economic downturns, and integrate health security planning within broader national development strategies. Such approaches are likely to improve resilience while simultaneously advancing progress toward Universal Health Coverage and the health-related Sustainable Development Goals [21,22].

The findings also highlight the importance of adopting systems-based approaches to resilience measurement and policy evaluation. Rather than focusing exclusively on preparedness indicators such as Joint External Evaluation (JEE) scores or International Health Regulations core capacities, policymakers should monitor broader determinants including governance effectiveness, financing sustainability, workforce resilience, institutional learning, and service continuity. These multidimensional indicators provide a more comprehensive understanding of resilience and may better inform policy decisions aimed at strengthening health systems against future epidemics and other complex public health emergencies [5,12,16].

### 4.5. Policy implications, strengths, limitations and future research

The findings of this study have important implications for health policy and health-system strengthening across Sub-Saharan Africa. The persistence of Low-Resilience countries and the relatively limited probability of rapid transitions to High Resilience suggest that strengthening health systems requires sustained, long-term investment rather than episodic responses during disease outbreaks. This observation aligns closely with the World Health Organization (WHO) Health Emergency Preparedness, Response and Resilience (HEPR) Framework, which emphasizes that resilient health systems should simultaneously strengthen emergency preparedness, maintain essential health services, and continuously adapt to emerging threats through institutional learning and multisectoral collaboration [27]. Likewise, the International Health Regulations (2005) advocate continuous development of core capacities for surveillance, laboratory services, risk communication, workforce development, and emergency coordination as essential components of global health security [21,22].

The positive associations observed between health expenditure, domestic health financing, and resilience highlight the urgent need for African governments to increase sustainable domestic investment in health. Although development partners continue to play an important role in supporting health-system strengthening, excessive dependence on external financing may compromise long-term sustainability because donor priorities frequently change in response to evolving global health emergencies. Consistent with the recommendations of the Africa Leadership Meeting Declaration on Increasing Domestic Health Financing, governments should strengthen domestic resource mobilization, improve public financial management, and protect health budgets during periods of economic uncertainty. Such investments should prioritize primary health care, disease surveillance, laboratory systems, digital health infrastructure, emergency preparedness, and health workforce development, which collectively form the foundation of resilient health systems [16,24,25].

The findings also reinforce the importance of strengthening regional collaboration across Africa. Infectious disease outbreaks frequently transcend national borders, making health-system resilience a regional rather than solely national concern. Africa CDC has increasingly emphasized coordinated surveillance, genomic sequencing, laboratory networking, emergency operations centres, and cross-border information sharing as essential strategies for strengthening continental health security. Similarly, the New Public Health Order for Africa advocates strengthening local manufacturing of medical countermeasures, expanding public health institutions, investing in workforce development, and increasing domestic financing to improve preparedness for future epidemics. The present findings provide empirical support for these continental initiatives by demonstrating that resilience depends upon sustained institutional capacity rather than isolated emergency interventions [24,25].

An important implication of this study concerns resilience measurement itself. Existing international monitoring frameworks frequently emphasize preparedness capacities through instruments such as the Joint External Evaluation (JEE), State Party Annual Reporting (SPAR), or the Global Health Security Index. While these frameworks provide valuable information regarding technical capacities, they may not fully capture the dynamic nature of resilience, particularly the ability of health systems to sustain essential services, adapt to prolonged crises, and recover following repeated shocks [13,27]. The multidimensional resilience index and longitudinal transition modelling adopted in the present study therefore offer a complementary approach that captures both the structural and temporal dimensions of resilience. Integrating such approaches into routine monitoring could enable policymakers to identify deteriorating resilience trajectories before they culminate in large-scale health emergencies [5,16,22].

This study possesses several notable strengths. First, it utilised a longitudinal panel covering twenty-five Sub-Saharan African countries over more than two decades, thereby providing one of the most comprehensive assessments of resilience trajectories within the region. Second, resilience was measured using a multidimensional composite index incorporating preparedness, response, recovery, financing, and health-system capacity rather than relying on a single indicator. Third, the combination of principal component analysis, first-order Markov state-transition modelling, and multinomial logistic regression provided complementary insights into resilience classification, temporal dynamics, and determinants. Finally, the study integrated multiple internationally recognized data sources, thereby enhancing comparability across countries and reducing dependence on any single surveillance system or database.

Despite these strengths, several limitations should be acknowledged. First, the ecological design limits causal inference because analyses were conducted at the country level rather than at the individual, facility, or district level. Consequently, the findings should not be interpreted as demonstrating causal relationships between the explanatory variables and resilience outcomes.40 Second, although internationally standardized databases were used, variations in reporting quality, data completeness, and surveillance capacity across countries may have introduced measurement bias. Such limitations are well recognised in multinational health-system research involving low- and middle-income countries. Third, several important determinants of resilience—including political leadership, institutional trust, corruption, community engagement, social cohesion, and governance effectiveness—could not be measured consistently across all countries and years because comparable longitudinal datasets remain limited. Previous resilience studies have identified these factors as important determinants of adaptive capacity, suggesting that future research should integrate governance and institutional indicators where feasible [21,22]. Fourth, although the resilience classification demonstrated good discriminatory ability, the thresholds used to define Low, Moderate, and High Resilience were study-defined and should therefore be interpreted within the methodological context of this analysis [10,11,26,27].

Future research should build upon the present findings in several ways. First, multilevel analyses incorporating subnational data could provide a more detailed understanding of resilience variations within countries. Second, qualitative investigations examining governance processes, institutional learning, and leadership could complement quantitative resilience indicators and provide deeper insights into mechanisms driving resilience transitions. Third, future studies should evaluate how implementation of the WHO HEPR Framework, Africa CDC’s New Public Health Order, and revised International Health Regulations influences resilience trajectories over time. Finally, as climate change, antimicrobial resistance, urbanization, and emerging zoonotic diseases increasingly interact to shape global health risks, future resilience assessments should adopt integrated One Health approaches that examine the interconnected resilience of human, animal, and environmental health systems [22,24,28,29].

Overall, the findings demonstrate that health-system resilience in Sub-Saharan Africa is dynamic, multidimensional, and strongly influenced by long-term structural investments in financing, governance, and institutional capacity. Sustainable improvements in resilience will therefore require coordinated national and regional strategies that simultaneously strengthen universal health coverage, emergency preparedness, and health security while promoting adaptive learning and continuous system transformation.

## References

[1] Nachega JB, Nsanzimana S, Rawat A, Wilson LA, Rosenthal PJ, Siedner MJ, et al. Advancing detection and response capacities for emerging and re-emerging pathogens in Africa. Lancet Infect Dis. 2023;23(5):e185–9. doi: 10.1016/S1473-3099(22)00723-X 36563700 PMC10023168

[2] Mboera LEG, Rumisha SF, Mfinanga SG. Control and prevention of communicable diseases in sub-Saharan Africa: A policy rethink. Onderstepoort J Vet Res. 2014;81(2):1–8. 10.4102/ojvr.v81i2.734 25004929

[3] Omoleke SA, Mohammed I, Saidu Y. Ebola viral disease in West Africa: A threat to global health, economy and political stability. J Public Health Afr. 2016;7(1):534. doi: 10.4081/jphia.2016.534 28299152 PMC5349256

[4] Tambo E, Adetunde OT, Olalubi OA. Re-emerging Lassa fever outbreaks in Nigeria: Re-enforcing “One Health” community surveillance and emergency response practice. Infect Dis Poverty. 2018;7(1):37. doi: 10.1186/s40249-018-0421-8 29703243 PMC5923006

[5] Karamagi HC, Titi-Ofei R, Kipruto HK, Seydi AB-W, Droti B, Talisuna A, et al. On the resilience of health systems: A methodological exploration across countries in the WHO African Region. PLoS One. 2022;17(2):e0261904. doi: 10.1371/journal.pone.0261904 35130289 PMC8820618

[6] Omoleke SA, Ajibola O, Ajiboye JO, Raji RO. Quagmire of epidemic disease outbreaks reporting in Nigeria. BMJ Glob Health. 2018;3(1):e000659. doi: 10.1136/bmjgh-2017-000659 29527352 PMC5841526

[7] World Health Organization. Measles and rubella strategic framework 2021–2030. Geneva: World Health Organization; 2021.

[8] Africa Centres for Disease Control and Prevention. Cholera situation report. Addis Ababa: Africa CDC; 2023.

[9] Kruk ME, Myers M, Varpilah ST, Dahn BT. What is a resilient health system? Lessons from Ebola. Lancet. 2015;385(9980):1910–2. doi: 10.1016/S0140-6736(15)60755-3 25987159

[10] Blanchet K, Nam SL, Ramalingam B, Pozo-Martin F. Governance and capacity to manage resilience of health systems: Towards a new conceptual framework. Int J Health Policy Manag. 2017;6(8):431–5. doi: 10.15171/ijhpm.2017.36 28812842 PMC5553211

[11] Thomas S, Sagan A, Larkin J, Cylus J, Figueras J, Karanikolos M. Strengthening health systems resilience: key concepts and strategies. Copenhagen: European Observatory on Health Systems and Policies; 2020. 32716618

[12] Organisation for economic co-operation and development, European Union, joint research centre. Handbook on constructing composite indicators: Methodology and user guide. Paris: OECD Publishing; 2008. doi: 10.1787/9789264043466-en

[13] Khalil M, Ravaghi H, Samhouri D, Abo J, Ali A, Sakr H, et al. What is “hospital resilience”? A scoping review on conceptualization, operationalization, and evaluation. Front Public Health. 2022;10:1009400. doi: 10.3389/fpubh.2022.1009400 36311596 PMC9614418

[14] Tumusiime P, Karamagi H, Titi-Ofei R, Amri M, Seydi ABW, Kipruto H, et al. Building health system resilience in the context of primary health care revitalization for attainment of UHC: proceedings from the Fifth Health Sector Directors’ Policy and Planning Meeting for the WHO African Region. BMC Proc. 2020;14(Suppl 19):16. doi: 10.1186/s12919-020-00203-2 33292240 PMC7710773

[15] Karamagi H, Titi-Ofei R, Amri M, Zombre S, Kipruto H, Seydi AB-W, et al. Cross country lessons sharing on practices, challenges and innovation in primary health care revitalization and universal health coverage implementation among 18 countries in the WHO African Region. Pan Afr Med J. 2022;41:159. doi: 10.11604/pamj.2022.41.159.28913 35573441 PMC9058992

[16] World Health Organization Regional Office for Africa. Framework for sustaining resilient health systems to achieve universal health coverage and promote health security, 2023–2030. Brazzaville: WHO Regional Office for Africa; 2023.

[17] Truppa C, Yaacoub S, Valente M, Celentano G, Ragazzoni L, Saulnier D. Health systems resilience in fragile and conflict-affected settings: a systematic scoping review. Confl Health. 2024;18(1):2. doi: 10.1186/s13031-023-00560-7 38172918 PMC10763433

[18] Haldane V, De Foo C, Abdalla SM, Jung A-S, Tan M, Wu S, et al. Health systems resilience in managing the COVID-19 pandemic: lessons from 28 countries. Nat Med. 2021;27(6):964–80. doi: 10.1038/s41591-021-01381-y 34002090

[19] Lal A, Erondu NA, Heymann DL, Gitahi G, Yates R. Fragmented health systems in COVID-19: rectifying the misalignment between global health security and universal health coverage. Lancet. 2021;397(10268):61–7. doi: 10.1016/S0140-6736(20)32228-5 33275906 PMC7834479

[20] Sachs JD, Karim SSA, Aknin L, Allen J, Brosbøl K, Colombo F, et al. The Lancet Commission on lessons for the future from the COVID-19 pandemic. Lancet. 2022;400(10359):1224–80. doi: 10.1016/S0140-6736(22)01585-9 36115368 PMC9539542

[21] World Health Organization. International health regulations (2005). 3rd ed. Geneva: World Health Organization; 2016.

[22] World Health Organization. Health emergency preparedness, response and resilience framework. Geneva: World Health Organization; 2022.

[23] Karamagi HC, Tumusiime P, Titi-Ofei R, et al. Towards universal health coverage in the WHO African Region: Assessing health-system resilience and primary health care performance. BMJ Glob Health. 2023;8(Suppl 7):e013122. doi: 10.1136/bmjgh-2020-004618 33789869 PMC8015798

[24] Africa Centres for Disease Control and Prevention. Africa CDC strategic plan 2023–2027. Addis Ababa: Africa CDC; 2023.

[25] Nkengasong JN, Tessema SK. Africa’s new public health order for a new era. Lancet Glob Health. 2023;11(5):e713–e714.

[26] Morgenstern H. Ecologic studies in epidemiology: concepts, principles, and methods. Annu Rev Public Health. 1995;16:61–81. doi: 10.1146/annurev.pu.16.050195.000425 7639884

[27] Boerma JT, Mathers C, Abou-Zahr C. WHO and global health monitoring: The way forward. PLoS Med. 2010;7(11):e1000373. doi: 10.1371/journal.pmed.1000373 21151348 PMC2994664

[28] World Health Organization, Food and Agriculture Organization of the United Nations, World Organisation for Animal Health, United Nations Environment Programme. One Health joint plan of action, 2022–2026. Geneva: World Health Organization; 2022.

[29] Destoumieux-Garzón D, Mavingui P, Boetsch G, Boissier J, Darriet F, Duboz P, et al. The one health concept: 10 years old and a long road ahead. Front Vet Sci. 2018;5:14. doi: 10.3389/fvets.2018.00014 29484301 PMC5816263

